# Short-Term Stability of Electrochemical Properties of Layer-by-Layer Coated Heterogeneous Ion Exchange Membranes

**DOI:** 10.3390/membranes13010045

**Published:** 2022-12-29

**Authors:** Veronika Sarapulova, Ekaterina Nevakshenova, Kseniia Tsygurina, Valentina Ruleva, Anna Kirichenko, Ksenia Kirichenko

**Affiliations:** 1 Physical Chemistry Department, Kuban State University, 149 Stavropolskaya st., 350040 Krasnodar, Russia; 2 Department of Electric Engineering Thermotechnics and Renewable Energy Sources, Kuban State Agrarian University Named after I.T. Trubilin, 13 Kalinina st., 350004 Krasnodar, Russia

**Keywords:** ion exchange membrane, membrane modification, layer by layer coating, heterogeneous membrane, voltammetry, i-V curve, stability of coating, polyethylenimine, polyallylamine, solution dipping

## Abstract

Layer-by-layer adsorption allows the creation of versatile functional coatings for ion exchange membranes, but the stability of the coating and resulting properties of modified membranes in their operation is a frequently asked question. This paper examines the changes in voltammetric curves of layer-by-layer coated cation exchange membranes and pH-metry of desalination chamber with a studied membrane and an auxiliary anion exchange membrane after short-term tests, including over-limiting current modes. The practical operation of the membranes did not affect the voltammetric curves, but enhanced the generation of H^+^ and OH^−^ ions in a system with polyethylenimine modified membrane in Ca^2+^ containing solution. It is shown that a distinction between the voltammetric curves of the membranes modified and the different polyamines persists during the operation and that, in the case of polyethylenimine, there is an additional zone of growth of potential drop in voltammetric curves and stronger generation of H^+^ and OH^−^ ions as indicated by pH-metry.

## 1. Introduction

The method of layer-by-layer assembly of coatings [1], which gained popularity in the 1990s, is used in membrane technologies for several effects including increase of the monovalent selectivity of membranes which is required, for example, in fractionation of Li^+^ from its mixture with Mg^2+^ [2,3] in brines of natural salt lakes and improvement of antifouling potential [4], which is especially important for anion exchange membranes used to generate energy from salinity gradient power [5]. In addition, layer-by-layer assembly is used also for biomedical purposes to create biomimetic coatings [6,7] and materials with a gradual release of substances [8].

Works that originally described the layer-by-layer assembly of materials from substances with different signs of charge were conducted on oxides (in particular, in [9], a chromatographic column was assembled from silica and alumina). In the 1990s the interest in the method was reignited by works in which polymers with alternating charges of polar groups were used [1]. Currently there are other approaches to creating layer-by-layer assembled coatings. For example, in coating, one of the types of layers can be made of a polymer and the second can consist of nanomaterials [10]. 

Layer-by-layer assembled materials can be divided into two categories by the requirements for their stability. The first category, consisting mainly of systems with controlled release of substances, is represented by materials with a planned destruction of the surface layer [11,12]. The second, which includes coatings with the main function of preserving food [13] or providing monovalent selectivity in separation processes [14], is represented by materials for which the absence of defects in coating is extremely important, since in the case of food the defects in the protective coatings can cause spoilage [15] and in the case of selective separation of ions the formed defects will serve as “bridges” for undesirable transport of doubly charged ions [16].

Such an influence of defects on the properties of the coating makes, in our experience, the stability of the system of layers formed on the surface of the membrane increase its selectivity; this is one of the most frequently asked questions, which is especially relevant in cases where the layers are not covalently crosslinked, but are assembled electrostatically or by another easily reversible mechanism (for example, by formation of hydrogen bonds). It is known that the layered assemblies may be destroyed due to harsh conditions, such as high ionic strength of treated solution [17] or presence of alkali [18]. We should note that these changes occurred during the long-term operation of the membranes and from the viewpoint of actual industrial application the evaluation of long term stability is more desirable. The most thorough analysis of the stability of ion exchange membranes and their properties would be when the change in properties is studied throughout the entire lifetime of the membranes, which can be years (see, for example, the study of ageing of ion exchange membranes in the food industry [19]). In the case of membranes fabricated or modified by the layer-by-layer assembly method, the analysis of such results is complicated by the rather weak introduction of such a membrane modification into industry at the moment (a review of layer-by-layer assembled nanofilms points out that “relatively few multilayer films have had widespread impact outside of research environments” [20]), which, apparently, leads to the fact that the bulk of the published data focuses on the modification procedure and characterization of novel membranes, and the preservation of the coating during operation and the resulting stability of its properties is a much more rarely covered topic.

At the same time, potential damage to the modifying layer can begin not only after several (or several hundreds) hours of operation of the membranes, but also already during the assembly of the installation. If the changes occur early during the operation of the membranes, it will cause shift of measured properties during the membrane characterization, making the values time dependent and, consequently, the average values would depend on the number of repeats, hindering the membrane characterization. Short-term operation measurements detect such changes and additionally help in determining a timeframe in which the difference in membrane properties begin to appear, and it was shown that the 50 h long operation of the layer-by-layer coated heterogeneous membrane, modifying layers one of which was polyethylenimine, in under-limiting current mode electrodialysis creates differences in the voltammetric curve of the membrane [21]. The changes in structure can be attributed to multiple causes including partial drying and swelling of the membrane leading to cracks and bends. In addition, during the operation, the membrane is affected by such factors as interaction with the components of the treated solution [22], changes in salinity (which in the long term lead to a change in the swelling of the membrane and, if the substrate membrane and the modifying layers possess unequal elongation in swelling, will also lead to the formation of cracks or creases), chemical reaction with H^+^ and OH^−^ ions generated at the interface and other factors. The presence of OH^−^ ions seems to be the most dangerous for stability of the coating since they are able to deprotonate amino groups, damage the membrane matrix if it contains PVC [23,24] and enter into other undesirable interactions [25], which in some cases even led to delamination of the layered coating [18]. Thus, for electrodialysis the most challenging operating mode is over-limiting current, which, although advantageous from a perspective of higher performance of the target salt ion mass transport process [26,27], is achieved through several mechanisms including enhanced development of electro-convective vortices [28] which allows the gaining of economic benefit through the reduction in area of expensive ion exchange membranes [26] and decrease in capital costs through reduced equipment sizes [28]; this is also distinguished by a more intensive generation of H^+^ and OH^−^ ions [29] which may cause deterioration of membrane properties. Interestingly, transition to over-limiting current can be beneficial for membrane stability from the aspect of fouling resistance, since it was demonstrated that peptide fouling is weakened at over-limiting currents presumably due to water splitting products repelling the peptide charges and due to electro-convective vortices washing the foulants out [30].

The question of stability of ion exchange membranes during operation is also important for other applications such as anion exchange membrane water electrolyzers [31], fuel cells [32] and redox flow batteries [33], where both high chemical stability and mechanical strength are required. One of the approaches attempted for fuel cells is the introduction of functionalized graphene oxide as crosslinking agent due to formation of the π-π bonds [34].

In this work, the i-V curves of layer-by-layer coated heterogeneous cation exchange membranes and the changes in pH between the outlet and the inlet of the desalination chamber formed by such membranes and an auxiliary anion exchange membrane are analyzed, before and after the short-term operation of the system at several currents including over-limiting currents. It is shown that the i-V curves insignificantly changed as a result of operation, indicating the general resistance of such type of coating to factors typical of electrodialysis, that the dependence of the shape of i-V curve on the nature of the modifier persists during the operation, and that for one of the modified membranes, operation in CaCl_2_ solution led to a notable increase of the generation of H^+^ and OH^−^ ions, which can be attributed to the damage of polyamine by the OH^−^ ions.

## 2. Materials and Methods

### 2.1. Membrane Preparation and Modification

#### 2.1.1. Materials for Preparation and Modification

To study the stability of the coating, two types of modified membranes were made, each of which consisted of only three components.

The first component was a commercially available heterogeneous MK-40 ion exchange substrate membrane (Shchekinoazot Ltd., Pervomayskiy, Russia), which in turn is made of powders of polyethylene and a copolymer of sulfonated styrene and divinylbenzene, hot pressed between two polyamide cloths. As a result, the surface of the MK-40 membrane is geometrically and chemically inhomogeneous, and its important properties for layer-by-layer coating are that the surface is approximately 80 percent polyethylene [35] and that there are macropores between the ion exchange grains and polyethylene, the characteristic diameter of which can reach hundreds of micrometers and can change significantly during swelling and drying. The manufacturer’s website provides the following information about these membranes (Table 1):

A review [37] also lists several properties of MK-40 such as its ion exchange capacity in a swollen state, which is listed as 1.7 ± 0.1 mM/cm^3^. 

Membrane conditioning required a solution of salts. The salts were purchased from Vekton, St. Petersburg, Russia, and distilled water was produced directly in the laboratory. It is known that the surface of these heterogeneous membranes can be homogenized by formation of a layer of perfluorinated sulfonic cation exchanger [38]; in this case, a sufficiently thick layer of the order of tens of microns is formed, leveling the inhomogeneities of the membrane. Based on the foregoing, prior to layer-by-layer adsorption of polymer layers from dispersions, a homogenizing layer of perfluorinated sulfonic cation exchanger was formed on the surface of a heterogeneous membrane, which became the second component of the membrane. In this work, a LF-4SC perfluorinated sulfonic cation exchanger (similar in structure to Nafion material) in isopropyl alcohol (dispersion was purchased in ready-to-use form from Plastpolymer, St. Petersburg, Russia) was used.

The third component was a single layer of polyamine adsorbed from the dispersion— in one case this was polyallylamine (purchased from Merck KGaA, Darmstadt, Germany; the resulting membrane is denoted as MK-40-M-PAH) and in the other case it was polyethylenimine (purchased from Merck KGaA, Darmstadt, Germany; the resulting membrane is denoted as MK-40-M-PEI). Polyallylamine is used for layer-by-layer assembly [39] as a component of new promising materials (such as thermo-responsive polymers with cleavage-induced phase transition [40] or coatings decomposing by application of electric potential [41]), including biomedical applications such as creation of nano-capsules for drug delivery [42] and food packaging [43]. Polyethylenimine is frequently used as a polymer carrier [44] and basis for loading of nanoparticles [45] or coating for medical applications [46]. However, its use in biomedical application is shaped by the cyto-staticity [47] or cytotoxicity of amine groups which simultaneously makes it non-biocompatible [48] and useful as antimicrobial and anticancer agent [49,50]. Both of these polyamines are popular components of layered coatings aimed to increase monovalent selectivity [14,51,52,53]. It was expected that possible damage to the modifying layer would have a stronger effect on the electrochemical characteristics of the membrane if this layer is the only one present, than in the case of the damage of the last layer among the multiple layers, and therefore such changes would be easier to detect. Stability of the MK-40 membrane modified with a higher number of layers of polymers in desalination of a mixed solution in under-limiting modes is discussed in our other article [21].

#### 2.1.2. Procedures of Membrane Preparation and Modification

The membranes were purchased in a dry state. Three 6×6 cm^2^ fragments were cut from the purchased sheet, and three samples were stepwise equilibrated with 20 mM NaCl (dry salt was purchased from Vekton, St. Petersburg, Russia) solution (which contains monovalent counterion to the membrane, Na^+^), while three other samples were stepwise equilibrated with 10 mM (20 mM equivalent) CaCl_2_ (dry salt was purchased from Vekton, St. Petersburg, Russia) solution (which contains divalent counterion to the membrane, Ca^2+^), starting from one day in periodically replaced concentrated solutions of salts to conduct the ion exchange, and then periodically replacing the solution with more dilution to achieve the desired concentration. The equilibration was considered finished when the electrical conductivity of the solutions prior to contact with a membrane and after several hours of contact became equal to each other; in both cases, the equilibration lasted for 7 days. 

It should be noted that the concentrations of external solution were much lower than the ion exchange capacity of the substrate membrane (20 mM equivalent versus about 1.7 M equivalent per [37]), so an existence of relatively high Donnan potential difference [54] was expected.

To form a layer of perfluorosulfonic cation exchanger, the membranes were removed from the solutions of salts, blotted with filter paper, fixed at the edges with adhesive tape inside the Petri dish (Vekton, St. Petersburg, Russia) so that a window of at least 3 × 3 cm^2^ in size was formed in the geometric center of the membranes, and 0.2 mL of 7.2% dispersion (*w*/*w*) of LF-4SC was distributed over the surface of this window. Then the membranes were left in air for 30 min to evaporate the isopropyl alcohol and to solidify the LF-4SC layer.

In this study, the number of layers bearing polar groups charged oppositely to the polar groups of the membrane is limited to one; membranes with a higher number of formed layers are discussed in our previous work [21]. Membranes with a single oppositely charged layer also possess monovalent selectivity and are used in fractionation of solutions [17], as well as the membranes with multiple layers. Membranes with a single layer charged oppositely to the membrane bulk also have the advantage as a model of layer-by-layer coated membranes in that possible damage to the modifying layer would have a stronger effect on the electrochemical characteristics of the membrane if this layer is the only one present, rather than in the case of damage of the last layer among the multiple layers, and therefore such changes would be easier to detect. The procedure described in [51] was taken as the basis for the layer-by-layer adsorption procedure. For formation of the top layer, a dispersion of polyamine with a concentration of 1 g/L in distilled water was prepared, 100 mL of the dispersion was poured inside a Petri dish which contained the membrane and was left to adsorb for 30 min, after which the dispersion was discarded; the membrane was washed three times with distilled water to remove the unadsorbed polymer, after which the sample was placed in a salt solution of choice, 20 mM NaCl solution or 10 mM (20 mM equivalent) CaCl_2_ solution, for a day. Composition of the resulting samples is given in Table 2.

### 2.2. Thickness Measurements

The thicknesses of the deposited layers were estimated by measuring the thickness of the swollen membranes before and after the layer was applied using an MKC-25 0.001 micrometer (Micron Ltd., Moscow, Russia). Its nominal accuracy is 0.1 microns. The thickness was measured in 10 repeats in a way that each repeat was at the center of a (relatively) equal area of the membrane surface. Then, for each case, the mean thickness was calculated, the difference of two mean thicknesses was taken as a layer thickness and the sum of the uncertainty margins of these two measurements was taken as an uncertainty of determination of the layer thickness. The confidence intervals were calculated using t-statistics for *p* = 0.05.

### 2.3. i-V Curves

**Figure 1:**
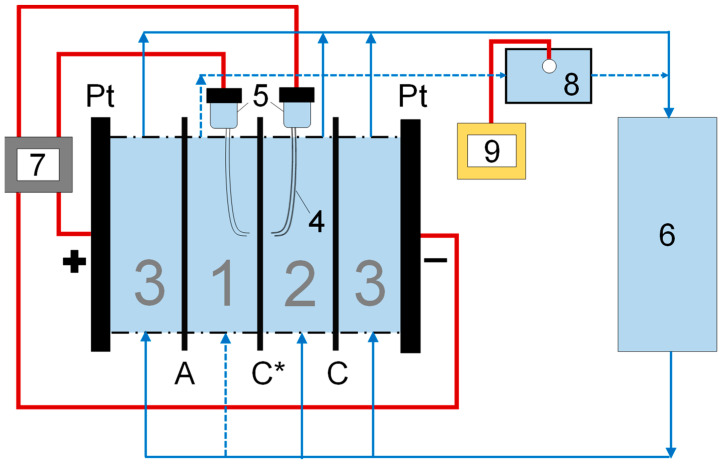
Scheme of the electrodialysis cell in the variant used for registration of i-V curves. 1 is the desalination chamber, 2 is the auxiliary chamber, 3 are the electrode chambers, 4 is the Luggin capillary, 5 is the Ag/AgCl electrodes, 6 is the common tank that holds glass electrode, 7 is the power supply and the voltmeter (united as the Autolab PGStat N100 in this case), 8 is the interstitial tank that also holds glass electrode and 9 is the pH meter. Dashed line shows the flow inlet and the outlet of the desalination chamber. The figure is an adaptation of the scheme from our previous article [55] (adapted under the terms of the Creative Commons Attribution 4.0 International License (http://creativecommons.org/licenses/by/4.0/, 20 November 2022)).

Studies of the stability of membrane properties were carried out as follows:

First, the i-V curves of newly prepared membranes were recorded in individual NaCl and CaCl_2_ solutions of the same equivalent concentration of 20 mM using a four-chamber flow-through cell for registration of the electrochemical characteristics (Figure 1).

The cell consists of two electrode chambers, an auxiliary chamber and a desalination chamber. The chambers are divided by three membranes: the membrane under study (in the center of the cell) and two auxiliary membranes separating the electrode chambers. One of the auxiliary membranes is the MK-40 membrane similar to that studied (but originating from the different sheet), and the second membrane is the MA-41 heterogeneous anion exchange membrane of the same manufacturer as the studied membrane, produced from powders of polyethylene and a copolymer of trimethyl-aminated styrene and divinylbenzene hot pressed between polyamide cloths. The solution is supplied to all four chambers from a common tank, and after passing through the cell, the solution is recirculated by a Heidolph Hei-FLOW Precision 01 multichannel peristaltic pump (Heidolph Instruments GmbH & Co. KG, Schwabach, Germany) back to the common tank. To record the i-V curves, the installation was filled with a salt solution and the solution flow rate was set, after which the Autolab PGStat N100 power supply-voltmeter (Metrohm, Utrecht, the Netherlands) set the stepwise current sweep between the polarizing electrodes in the range from 0 to 5 mA/cm^2^ at a rate of 2.5×10^−3^ mA/(cm^2^s). To determine the relative intensity of the generation of H^+^ and OH^−^ ions at the surface of the cation exchange and the paired anion exchange membranes forming the desalination chamber, glass electrodes connected to an Expert 002 pH meter (Econics-Expert, Moscow, Russia) were placed in a common recirculation tank and in an interstitial tank located at the outlet of the desalination chamber.

### 2.4. Operation under Direct Current

After recording the i-V curves, the polarizing current was switched off and the solution was pumped through the cell without application of current for 10 min. Then, a constant polarizing current was set in the system, the value of which increased from 0.75 theoretical limiting to 1.75 theoretical limiting. First, a direct current density was set in the system equal to 0.75 theoretical limiting one for 600 s, after which the polarizing current was turned off for 220 s. Next, the current density was set equal to 1.00 limiting for 600 s and a pause was made for 220 s, then the current density was set to 1.25 limiting ones for 600 s and a pause was made for 220 s, then the current density was set to 1.50 limiting ones for 600 s and a pause was made for 220 s, and finally the current density was set to 1.75 theoretical limiting ones and paused for 220 s. The theoretical limiting current density was calculated by the Lévêque equation (Equation (1)) [56].

(1) ilimtheor=zkCkFDhT1−t11.47h2V0LD13−0.2

where *z_k_C*_k_ is 2 × 10^−5^ mol/cm^3^ for both salts, *F* is the Faraday constant (9.6485×10^6^ mA × s/mol), *D* is the salt diffusion coefficient in the solution (assumed to be equal to the salt diffusion coefficient of the infinitely dilute solution, 1.61 × 10^−5^ cm^2^/s for NaCl and 1.34 × 10^−5^ cm^2^/s for CaCl_2_, respectively); *h* is the intermembrane distance (0.59 cm); *T*_1_ and *t*_1_ are the counterion transport numbers in the membrane and in the solution, respectively (the membrane was assumed to be absolutely selective, hence *T*_1_ is taken as 1, *t*_1_ was 0.603 for Na^+^ in NaCl and *t*_1_ was 0.438 for Ca^2+^ in CaCl_2_); *V*_0_ is the linear solution flow rate (0.36 cm/s); *L* is the length of the desalination path (2.15 cm).

For a 20 mM NaCl solution, the calculated theoretical limiting current density is 1.96 mA/cm^2^ and for a 10 mM (20 mM) equivalent CaCl_2_ solution this is 1.86 mA/cm^2^.

Note that the Lévêque equation was derived for the case of a homogeneous single-layer membrane and in the present study the calculated theoretical current density is used, rather, as a unifying benchmark. Equations for calculating the limiting current densities of multilayer membranes were derived in [57].

Experimental limiting current densities can be determined by various graphical methods [58], for example, using the method of finding the intersection of tangents drawn to the so-called ohmic region of the i-V curves and to the plateau section [59].

After the operation under direct current, another pair of i-V curves was recorded for each membrane, one in 20 mM NaCl solution and one in 10 mM CaCl_2_ solution. Note that the setup was not disassembled and hence the distance between the capillaries did not change from the beginning and until the end of all measurements made in one solution.

### 2.5. Attenuated Total Reflection Fourier Transform Infrared Spectroscopy

As an example of spectroscopic detection of changes occurring during membrane operation, we provide attenuated total reflection Fourier transform infrared spectra of MK-40 membrane and two series of membranes produced by the similar technique that operated in electrodialysis of mixed solution (15 mM NaCl and 7.5 mM CaCl_2_) for 50 h in under-limiting current mode (approximately 0.5 theoretical limiting values calculated by the approach described in [60]). Membrane preparation and operation are described in [21]; in brief, the membranes were also based on MK-40 and underwent surface homogenization with LF-4SC layer and layer-by layer absorption of three layers total: two of polyamine (polyallylamine orpolyethylenimine, depending on series) and one middle layer of sodium polystyrene sulfonate (purchased from Merck KGaA, Darmstadt, Germany). The ATR-FTIR spectra were registered by the “Diagnostics of structure and properties of nanomaterials” Centre for Collective Use of Kuban State University using a Bruker Optics Vertex 70 spectrometer (Bruker, Billerica, MA, USA) for newly prepared membranes and for membranes after operation under current; the measurement were made for dry membranes.

## 3. Results and Discussion

### 3.1. Thickness of Formed Layers

Thickness measurement of newly prepared samples are given in Table 3. The confidence intervals are calculated by t-statistics for *p* = 0.05. The values of membrane thicknesses measured after their operation lay within the uncertainty margin of the initial measurement.

We suppose that the large uncertainty margins, exceeding sometimes the average values, are at least partially the consequence of the use of differential methods of calculation of layer thicknesses, since the absolute errors add up in subtraction, and of the compressibility of swollen polymeric layers by faces of the micrometer. Additional reasons might be found through comparison with the results obtained by scanning electrical microscopy of a similar membrane with a larger number of adsorbed layers (differing by addition of layer of polystyrene sulfonate, PSS and polyamine) [21]. Scanning electron microscopy showed that the thickness of the coating differs by the coordinate at the membrane, presumably due to filling of the geometry of heterogeneous membrane by the modifying layers, and gives the thickness of assembly of LF-4SC + PAH + PSS + PAH of 5.12 ± 2.96 μm and the thickness of assembly of LF-4SC + PEI + PSS + PEI of 8.89 ± 7.25 μm. These results show that the large confidence intervals persist even without the differential method and without the use of micrometer and demonstrate that, while the thickness of coating is in the scale of micrometers, it has high intrinsic variation.

### 3.2. Voltammetric Curves

Voltammetric curves and pH-metry data for newly made modified membranes were previously reported in [61] and are used as comparison to illustrate the changes occurring as a result of membrane operation. The voltammetric curves and pH-metry of membranes after the operation are reported for the first time.

**Figure 2:**
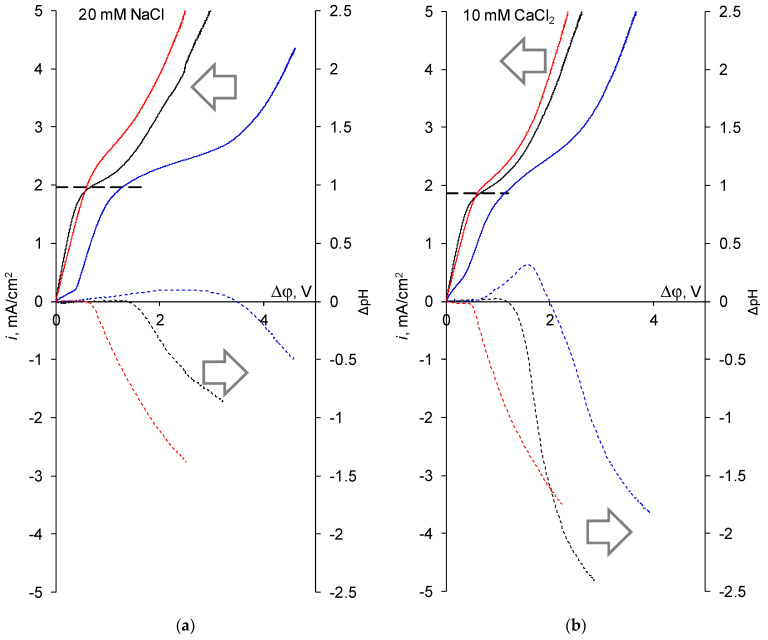
Registered in 20 mM NaCl solution (**a**) and in 10 mM CaCl_2_ solution (**b**) i-V curves (solid lines) and pH differences between the outlet and inlet of the desalination chamber (dashed lines) of the MK-40 substrate membrane (black lines), MK-40-M-PEI membrane (blue lines) and MK-40-M-PAH membrane (red lines).

Figure 2 shows a comparison of the i-V curves of layer-by-layer coated membranes recorded immediately after the formation of the modifying layers, i-V curve of the substrate membrane used as a control and the corresponding pH differences between the outlet and the inlet of the desalination chamber recorded in a NaCl solution and in a CaCl_2_ solution.

It can be seen that the i-V curve of the MK-40-M-PEI membrane has the initial segment of the growth of the potential drop both in the NaCl solution and in the CaCl_2_ solution, which we attribute to the barrier effect of this membrane towards the transport of any cation through the polyethylenimine layer. Note that in this case the section of the rapid growth of the potential drop is even more pronounced for the curve recorded in a solution containing a monovalent cation than in a solution containing a polyvalent cation, which suggests that the presence of such a section will not be associated with an increased monovalent selectivity.

It can also be seen that the curves recorded for the MK-40-M-PAH membrane have a shape typical of monopolar membranes (similar, for example, to the curves from [62]), except for a slightly higher limiting current density, which indicates that the ion transport through this membrane will be similar to the transport of ions through the substrate membrane.

Considering the pH shifts when the solution passes through the desalination chamber formed by a studied cation exchange membrane and the same paired anion exchange membrane, the differences can be noted between the two modified membranes. In the case of the MK-40-M-PAH membrane the pH-metry curve shifts to the low pH region in comparison with the curve registered for MK-40. Taking into account the same paired membrane this indicates that the generation of H^+^ and OH^−^ ions at the cation exchange membrane is weakened, possibly due to expansion of the “funnel” leading to a conductive zone framed by nonconductive ones due to application of a homogenizing material [63] and the subsequent decrease in the concentration polarization, which in turn increases the concentration of salt ions near the surface and hence delays the initiation of intensive generation of H^+^ and OH^−^ ions [64]. The effect of homogenization can also explain the increase in the limiting current density. In the case of the MK-40-M-PEI membrane, the pH-metry curves are significantly shifted to the alkaline region, which indicates an increase in the generation of H^+^ and OH^−^ ions at the surface of this membrane. It should be noted that, despite the significant shift of the curve as a whole, the values of the pH shift in under-limiting current modes changed rather weakly as a result of this modification, concluding that, despite the intensive occurrence of the side reaction of generation of H^+^ and OH^−^ ions in over-limiting current modes which reduces the current efficiency in the electrodialysis in galvanostatic mode and causes chemical destruction of the membrane, the membrane can be used if its operation is restricted to the under-limiting currents.

**Figure 3:**
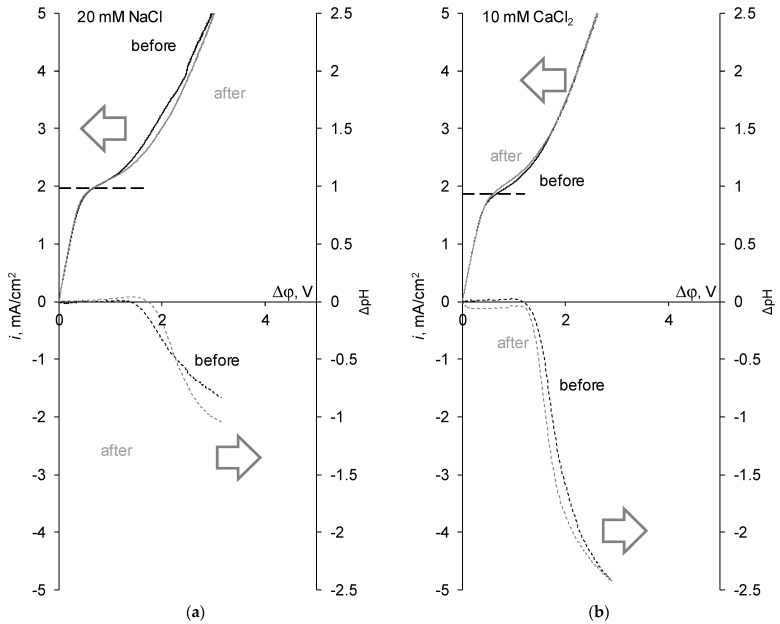

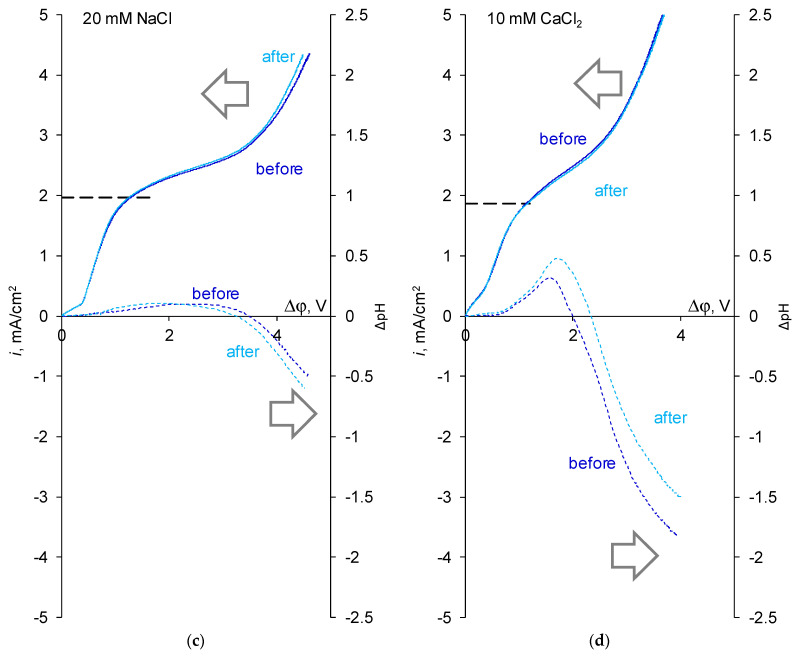

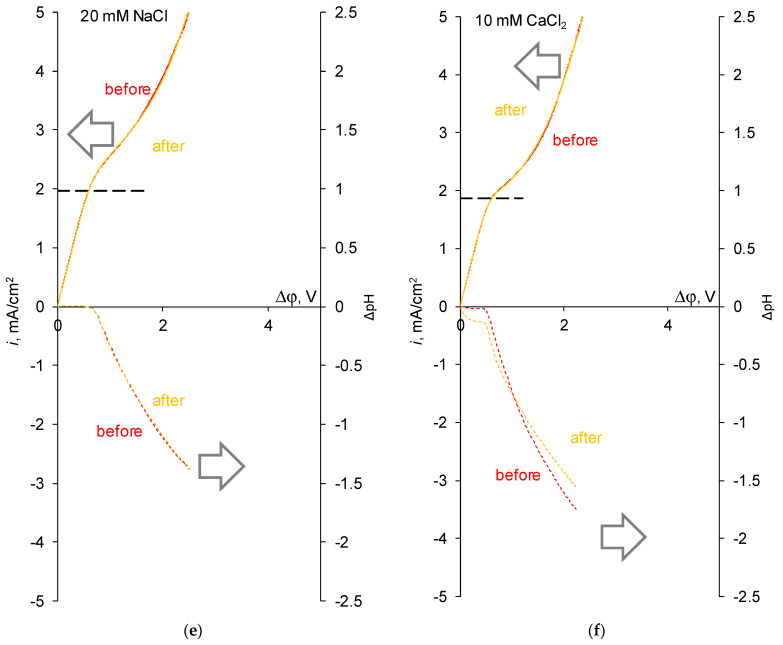
Recorded in 20 mM NaCl solution (**a**,**c**,**e**) and in 10 mM CaCl2 solution (**b**,**d**,**f**), i-V curves of the MK-40 substrate membranes (**a**,**b**), MK-40-M-PEI membranes (**c**,**d**) and MK-40-M-PAA membranes (**e**,**f**) (solid lines), and pH differences between the outlet and inlet to the desalination chamber (dashed lines). Black horizontal line with long dashes shows the theoretical limiting current density calculated by the Equation (1).

Figure 3 shows a comparison of the i-V curves of the membranes and the pH-metry of the outlet and the inlet of the desalination chamber before and after short-term operation of the experimental setup with installed membranes under current.

It can be seen that the i-V curves almost completely coincide, which is especially noticeable in the case of the modified MK-40-M-PAH membrane. This allows a conclusion that, despite that in this study the operation included the use of over-limiting current modes, in the duration of the study this did not lead to damage to the modification layer that affects the resistance (slope of the initial, so called ohmic, region of the i-V curve), the limiting current density or the ease of transition to the over-limiting current modes. In the case of pH-metry, it can be noted that the curves of some membranes have shifted to lower pH, which may be associated with degradation of the paired anion exchange membrane, namely the transformation of its polar groups, quaternary ammonium bases, into amino groups that are more active in the catalytic reaction of generation of H^+^ and OH^−^ ions [65]. The degradation of quaternary ammonium bases of the desalination chamber side of Neosepta AMX anion exchange membrane, operated in desalination of NaCl solution in over-limiting current modes, was confirmed by FTIR in [66], the degradation of the anion exchange side of membrane submerged in concentrated alkali was proven by FTIR in [67], and the degradation of MA-41-type membrane and Ralex AMH membranes immersed in alkali was shown by complexation of the formed amines with Cu^2+^ ions followed by atomic adsorption spectroscopy in [68], which may serve as evidence of the existence of such a mechanism; however, cited references describe much harsher conditions, usually much higher preexisting concentration of alkali, than those used in the present article. The pH-metry curve shifted significantly into the alkaline region due to operation in the only case of the MK-40-M-PEI membrane in a CaCl_2_ solution. It can be assumed that this is caused by the initially intensive generation of H^+^ and OH^−^ ions in this system providing OH^−^ ions that can damage the amino groups of the polyethylenimine.

The fact that membrane operation changed the pH-metry curves far more than it changed the i-V curves raises the question whether the pH-metry data are more suitable for earlier detection of the degradation of the chemical composition of the membranes than the i-V curves. Answering this question would require a longer stability study that would periodically register and compare the i-V curves and the pH-metry curves of membrane systems.

### 3.3. Considerations on the Possible Changes in Composition Based on ATR-FTIR Spectra

**Figure 4:**
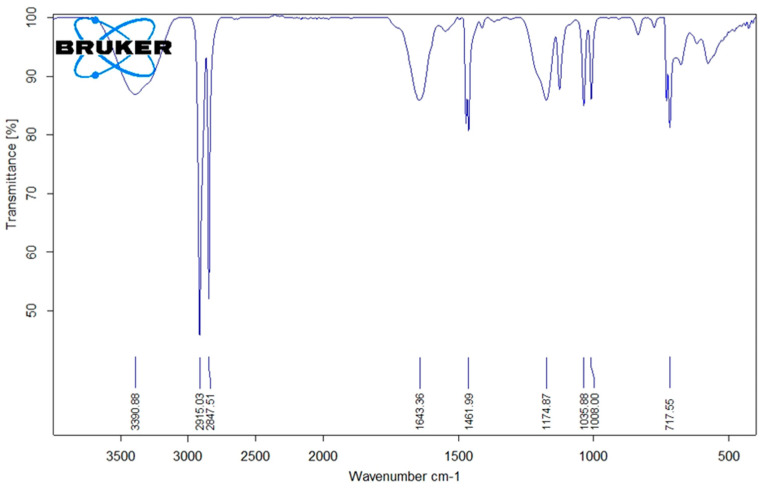
ATR-FTIR spectrum of MK-40 membrane.

FTIR spectra of the MK-40 membrane (Figure 4) confirm that its surface is mostly covered with polyethylene, as evidenced by the bands at 2915 cm^−1^, 2848 cm^−1^, 1462 cm^−1^ and 718 cm^−1^ (Bruker website has an example of a polyethylene spectrum with bands at 2915 cm^−1^, 2848 cm^−1^, 718 cm^−1^ and a peak at 1470 cm^−1^ recommended for its determination). The band at 1036 cm^−1^ might be attributed to SO_3_H group.

**Figure 5:**
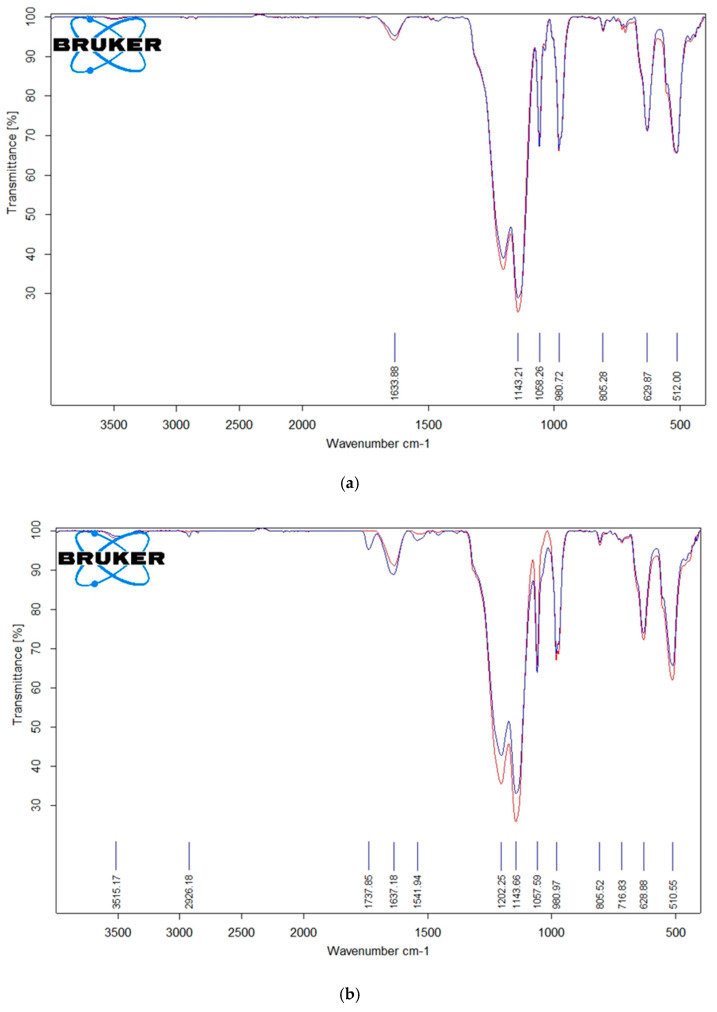
ATR-FTIR spectrum of layer-by-layer coated membranes which contained polyallylamine (**a**) or polyethylenimine (**b**) before (blue line) and after (red line) membrane operation under direct current.

Coating of the membrane with layers of polymers almost completely eliminates these bands. The signs of presence of sulfonic groups, however, becomes much more noticeable in spectra of modified membranes (it should be noted again that the spectra given below correspond to the membranes of similar composition but not precisely to the studied membranes, for example, the membranes’ spectra were registered as having a sodium polystyrene sulfonate layer and were operating under direct current of constant value for 50 h). Figure 5 gives the ATR-FTIR spectra of layer-by-layer coated membranes that contained polyallylamine (Figure 5a) and polyethylenimine (Figure 5b). Despite the topmost layer of the membranes being composed of polyamine and the sodium polystyrene sulfonate being only the second from the top, the spectra show very strong bands at 1202 cm^−1^ and 1143 cm^−1^, which are attributed to the SO_3_H group.

The bands attributed to amine groups are at 1637 cm^−1^ (attributed to N-H vibrations of secondary amines) and 1542 cm^−1^ (attributed to N-H vibrations of primary amines [69]).

It can be seen that the spectrum of the membrane that contained polyethylenimine experienced the stronger change and that the changes in intensity of bands attributed to polar groups occur in two directions: bands attributed to the presence of the topmost layer get weaker while the bands attributed to the presence of the second from the top layer get stronger, which may indicate the damage to the coating.

## 4. Conclusions

Layer-by-layer modification of a heterogeneous ion exchange membrane leads to a change in its electrochemical characteristics, the direction of which depends on the nature of the modifier. If a cation exchange membrane is coated with homogenizing cation exchange layer and polyallylamine as anion exchange layer, then the effect of surface homogenization is decisive and the limiting current density through the membrane increases while the generation of H^+^ and OH^−^ ions is suppressed. If a cation exchange membrane is coated with a homogenizing layer and a polyethylenimine as an anion exchange layer, then the barrier effect of polyethylenimine towards the transport of both monovalent and polyvalent salt cations becomes decisive, and the potential drops in the i-V curves increase while the generation of H^+^ and OH^−^ ions at this membrane is boosted.

The operation of membranes under current, which included the stages in over-limiting current modes, did not lead to a significant change in the i-V curves, which can be interpreted as evidence of the resistance of the fabricated layer-by-layer modified membranes to external factors, typical of the electrodialysis process. The changes in pH-metry of the majority of membrane-solution systems are attributed not to the processes related to the studied membrane but to degradation of paired anion exchange membrane. However, as a result of the operation of the membrane modified with polyethylenimine in a 10 mM CaCl_2_ solution, the generation of H^+^ and OH^−^ ions significantly increased, which can be caused by the starting intensive generation of H^+^ and OH^−^ ions in this system that led to the damage of the polyethylenimine. This finding raises the question of sensitivity of pH-metry in comparison with voltammetry for early detection of changes in membrane composition.

## Figures and Tables

**Table 1:** Properties of the MK-40 membrane declared by the manufacturer [36] (translated from Russian).

Property	Value
Thickness, mm	0.3–0.5
Elongation during swelling, %, in length	8 ± 2
Elongation during swelling, %, in thickness	30 ± 5
Area resistance, Ohm·cm^2^, no more than	10.0
[counterions] transport number, fraction, no less than	0.8
Ion exchange group	sulfonic (SO_3_H)
Inert filler	polyethylene
Reinforcing cloth	polyamide

**Table 2:** Composition of created samples.

Sample	MK-40	MK-40-M-PAH	MK-40-M-PEI
Substrate	MK-40	MK-40	MK-40
Homogenizing layer	-	LF-4SC	LF-4SC
Outer layer	-	PAH	PEI
Tested in 20 mM NaCl	+	+	+
Tested in 10 mM CaCl_2_	+	+	+

**Table 3:** Calculated thickness of layers.

Layer Thickness	In 20 mM NaCl Solution	In 10 mM CaCl_2_ Solution
LF-4SC	2.4 ± 2	2.0 ± 2
PEI	11.8 ± 4	12.0 ± 5
PAH	2.4 ± 2.5	1.6 ± 2

## Data Availability

The data presented in this study are available on request from the corresponding author.
